# Subjective memory complaints of patients and caregivers and their association with sociodemographic and clinical factors in patients with neurocognitive disorders from Colombia

**DOI:** 10.21203/rs.3.rs-7040277/v1

**Published:** 2025-07-14

**Authors:** Felipe Botero-Rodríguez, Juan Camilo Castro, Salomón Salazar-Londoño, Juanita Moreno-Contreras, Maria José Bornacelly, Patrick Verhelst, José Manuel Santacruz-Escudero, Hernando Santamaría-García

**Affiliations:** Hospital Universitario San Ignacio; Hospital Universitario San Ignacio; Pontificia Universidad Javeriana; Pontificia Universidad Javeriana; Pontificia Universidad Javeriana; Hospital Universitario San Ignacio; Hospital Universitario San Ignacio; Hospital Universitario San Ignacio

**Keywords:** Subjective Cognitive Decline, Neurocognitive Disorders, Neuropsychiatric Symptoms, Geriatric Assessment, Neuropsychological Tests

## Abstract

Subjective memory complaints (SMC) are common in older adults and may signal cognitive decline. We examined the clinical correlates of patient-reported SMC and caregiver-reported complaints (CMC) and introduced their discrepancy (SMCΔ) as a potential marker of anosognosia or caregiver overestimation. We carried out a cross-sectional study and systematically assessed cognitive, functional, and behavioral domains of 6,708 individuals from a Latin American memory clinic. CMC scores were significantly higher than SMC and were more strongly associated with cognitive and functional impairments, while SMC was linked to neurological comorbidities and higher education. Psychiatric symptoms were associated with both CMC and SMCΔ. Lower cognitive scores predicted greater CMC, and SMCΔ was best explained by education, MoCA, and NPI-Q. Our findings suggest that SMC, CMC, and their discrepancy capture distinct aspects of disease awareness and caregiver perception, supporting the clinical utility of SMCΔ in early detection and assessment of neurodegenerative conditions.

## Introduction

Cognitive complaints serve as critical clinical markers associated with various neuropsychiatric conditions in older adults.^1^ Subjective memory complaint (SMC) is a patient-reported outcome measure with a prevalence that oscillates between 25% and 50% in people over 65 years old.^2^ SMC is one of the most frequently reported concerns in clinical settings and can be assessed without specialized training.^3,4^ It has been recognized as a marker for cognitive decline,^5^ as it is associated with memory functioning and brain markers, including structural,^6^ functional, and molecular ones.^7^ SMC may be influenced by multiple factors, including advanced age, lower education, affective status, stress, insomnia, and pain, as well as being most frequently reported by women.^7–9^

SMC are the first step in studying Alzheimer’s Disease (AD) according to the Latin American Task Force for Diagnosis.^10^ In dementia, caregiver cognitive complaints are also important, as their reports on patient’s cognition and behavior may help differentiate AD from other neurocognitive disorders such as frontotemporal dementia (FTD). In AD, caregiver memory complaints (CMC) are higher than in FTD, while in this latter condition caregiver complaints are mostly related to behavioral disturbances.^11^ Evidence suggests that integrating both patient- and caregiver-reported SMC may improve screening accuracy in neurodegeneration.^5^ Often, discrepancies arise between SMC and CMC,^5^ largely due to the patient’s anosognosia, common in individuals with relevant cognitive decline in AD.^11^ Both SMC and CMC have shown to be correlated with objective memory failures.^5,12,13^

Current evidence lacks a detailed characterization of the population reporting SMC and CMC and their associated factors. Moreover, a key challenge remains in refining methods to assess and differentiate SMC from CMC. While some studies have examined the clinical implications of SMC, a comprehensive analysis integrating clinical, cognitive, and socio-behavioral factors associated with SMC and CMC is still lacking, as well as the incorporation of both patient and caregiver perspectives.^14^ Examining sociodemographic variables, comorbidities, neuropsychiatric symptoms, cognitive performance, and functional status will provide deeper insights into the clinical and diagnostic role of SMC and CMC in neurodegenerative diseases.

This study aims to evaluate the sociodemographic and clinical correlations of both SMC and CMC, while also analyzing the discrepancy (SMCΔ) between them as a potential marker of anosognosia or caregiver overestimation. A higher SMCΔ, driven by greater SMC scores, may indicate heightened self-awareness of cognitive difficulties, whereas a lower SMCΔ, reflecting diminished SMC relative to CMC, may suggest anosognosia. To achieve this, we conducted a cross-sectional study using a sample (n = 3,354 patients and their caregivers, for a total of 6,708 individuals) from a memory clinic in Colombia, an underrepresented Latin American country in cognition and dementia research. We measured SMC and CMC, and gathered data to fully describe the profile of individuals based on sociodemographic factors and assessed a set of associated factors including cognitive performance, functional capacity, physical state, and neuropsychiatric symptoms.

We hypothesize that increased SMC and CMC will be associated with objective cognitive and functional impairments but with varying relationships depending on the severity of neurocognitive disorders. Specifically, we expect SMC to be higher in mild cases of cognitive decline as individuals recognize their difficulties, while it may decrease in advanced stages.^11^ Conversely, CMC is expected to increase in individuals with intermediate and high severity, as caregivers become more aware of deficits and more attuned to sensing and tracking changes, particularly in later stages of the condition. Our findings may contribute to the earlier detection of cognitive decline and enhance clinical tools for assessing neurocognitive disorders in aging populations.

## Methods

We carried out an observational analytical cross-sectional study. The research protocol was approved by the Research Institutional Ethics Committee of San Ignacio Hospital University and the Pontificia Universidad Javeriana, act N° 18/2016.

### Population and clinical assessment

**Figure 1:**
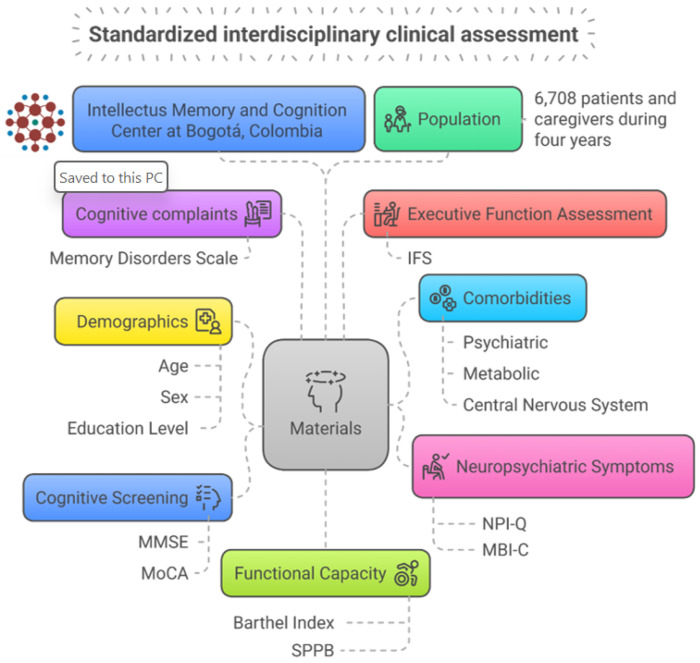
Overview of the standardized interdisciplinary clinical assessment held at Intellectus Memory and Cognition Center.

Our population consisted of 3,354 patients and their caregivers (n = 6,708) who attended at Intellectus Memory and Cognition Center of Hospital Universitario San Ignacio, Bogotá, Colombia, between January 1, 2018, and December 31, 2020. Patients were evaluated using a standardized interdisciplinary clinical assessment that includes geriatricians, psychiatrists, neurologists, and neuropsychologists. Furthermore, an interdisciplinary meeting was held to reach a consensus diagnosis and come up with appropriate management suggestions. This approach has been previously reported and validated as highly accurate in the diagnosis process.^15^ See Fig. 1.

### Materials

We used the Memory disorders scale to evaluate SMC and CMC, which had been validated in the Colombian population by Cano et al.^16^ The instrument has 15 items (Annex 1). The same battery was applied to the patient and the caregiver separately. Each question was answered based on the frequency of the complaint using a Likert scale (never, rarely, sometimes, always) having a 0–3 value. The maximum value is 45 and the minimum is 0, with a cutoff point of 19.

### Demographics and comorbidities:

Demographic assessment: The patient’s age and sex were based on informant and self-report (male or female). Education level was assessed based on years of schooling as a continuous variable.

### Cognitive screening:

The Mini-Mental State Examination (MMSE): This instrument was used to assess cognitive domains. MMSE has accurate psychometric characteristics, with 88.3% for sensibility and 87% for specificity. It evaluates cognitive domains, such as memory, language, executive function, and visuo-constructional capacity in people with neurocognitive disorders.^17,18^ MMSE has been validated in the Colombian population with sensitivity and specificity of 92.3 and 53.7%, respectively.^19^

Montreal Cognitive Assessment (MoCA): This instrument was used to detect more fine-grained cognitive functioning considering its properties in better capturing executive functioning. MoCA has a cut-off point of 26 points, and it consists of 19 questions, which assess eight domains (executive skills, naming, memory, attention, language, abstraction, delayed memory, and orientation).^20^ It has been used worldwide as a useful instrument for screening neurocognitive disorders, with diagnostic usefulness in detecting major neurocognitive disorders^21^. It was validated in the Colombian population demonstrating a sensibility of 76% to detect MCI and 92.7% to detect mild dementia, and a specificity of 79.8%.^22^

### Executive function assessment

The Ineco Frontal Screening (IFS): It was used to test executive functioning. It is composed of 7 domains: response inhibition and control over task change, the ability to respond appropriately to conflicting instructions, the ability to abstract, through the interpretation of proverbs, and working memory. Other studies have used this tool with patients diagnosed with different types of neurocognitive disorders to assess executive function.^23^ This scale has been validated in the Colombian population with an optimal cutoff of 17.5 with a sensitivity of 92.8% and a specificity of 86.3%.^24^

### Functional capacity and physicial performance:

Barthel index: this instrument was used to assess the patient’s independence in basic daily activities. This scale assesses ten activities of everyday life. The maximum score is 100 points, and the minimum is 0. It is also helpful for patient longitudinal follow-up.^25^ Barthel index has been validated in Spanish, reflecting good reliability and structural validity, and Cronbach’s alpha coefficients greater than 0.70.^26^ Short physical performance battery (SPPB): it was used to assess muscular function, by evaluating balance, gait, and strength/endurance. The first one is established by examining the ability to stand with the feet together in the side-by-side, semi-tandem, and tandem positions. The second one depends on the seconds to walk 8 feet. The third one is based on the time to rise from a chair and return to the seated position 5 times.^27^ SPPB is reliable and valid for assessing physical performance among older adults in Colombia, with a total test reliability of 0.87.^28^

### Neuropsychiatric symptoms assessment

Neuropsychiatric Inventory Questionnaire (NPI-Q): It was used to assess chronic neuropsychiatric symptoms in people with dementia. It comprises twelve domains (delusions, hallucinations, aggressiveness, depression, anxiety, euphoria, apathy, disinhibition, irritability, motor disturbance, nocturnal behavior, and appetite), which evaluate the presence and severity of the symptoms (1 = mild, 2 = moderate, 3 = severe). The maximum score is 36 points.^29^ NPI-Q has been validated in Spanish, showing a test-retest reliability of 0.89, and a convergence validity of 0.88.^30^

Mild Behavioral Impairment – Checklist (MBI-C): it was used to assess subtle neuropsychiatric symptoms. Previous studies have used it to assess mild behavioral impairment, having effect in the prediction of dementia risk.^31^ This scale arose from behavioral symptoms manifested in the early stages of FTD behavioral variant, for which the construct of mild behavioral impairment (MBI) was proposed.^32^ The MBI criteria states that the symptoms must be present for at least six months. Previous studies have demonstrated that it predicts other types of dementia and helps detect cognitive, neuropsychiatric, and functional symptoms. It comprises five domains (interest, affective symptoms, disinhibition, social norms, and delusions/hallucinations), and evaluates the presence and severity of symptoms on a Likert scale (1 = mild, 2 = moderate, 3 = severe).^31^ It is described to have a sensitivity of 1 and a specificity of 0.96 in patients with subjective decline.^33^ MBI-C has been translated into Spanish.^34^

### Comorbidities:

A medical history was retrieved, asking for all types of comorbidities, including psychiatric diagnosis, stroke, etc. Then, in our database, diseases were classified based on their nature, as a psychiatric, metabolic, or central nervous system comorbidity.^35^

## Statistical analysis

We conducted a descriptive analysis of SMC and CMC variables as well as, all factors associated. We followed this procedure to explore the population distribution and characteristics, as well as search for missing data and outliers. Then, we evaluated mean and proportion differences of SMC and CMC using the *student t-test* with the subsequent standard deviation and *test of equal or given proportions* between neurocognitive disorders at different stages, major and mild, as well as mild neurocognitive disorder and normal cognition. Afterwards, we evaluated possible collinearity between the variables analyzed using Spearman’s correlation based on the distribution of the variables, since it is less sensitive to outliers.^36^

Secondarily, to explore the association between SMC and CMC with sociodemographic and clinical factors, as well as the neuropsychiatric patterns in individuals, we defined three different outcomes: SMC, CMC and SMCΔ. SMCΔ was established by the subtraction of SMC minus CMC aiming to mitigate possible bias as stated in the introduction. We ran multiple linear regression models for each outcome in all the population and stratified by severity. We included as independent variables age, sex, metabolic disease, neurologic comorbidity, psychiatric comorbidity, Barthel index, SPPB, MoCA, MMSE, NPI-Q, MBI-C, and IFS. The p-value coefficients and McFadden’s R-squared (R2) were used to test the models. Subsequently, we selected the final independent variables in each model using backward elimination that selects the most parsimonious model considering the Akaike Information Criterion (AIC).

Finally, we constructed multiple additive risk models using logistic regression to estimate the association with the presence of SMCΔ ≥ 1 (considered a clinical proxy of patient overestimation) compared to SMC < 1 (considered a clinical proxy of caregiver overestimation) (dependent variable). This approach aligns with our objective of establishing a secondary measure for SMC while minimizing the potential effects of anosognosia or overestimation of cognitive impairment. The cut-off of 1 was chosen due to the nature of the difference, as subtraction effectively highlights the primary contributing factor (SMC or CMC). As independent variables, we included all previously established variables from prior models, followed by a backward elimination procedure to select the final model based on the lowest AIC. All analyses were conducted using R Studio version 4.2.1.

## Results

**Figure 2:**
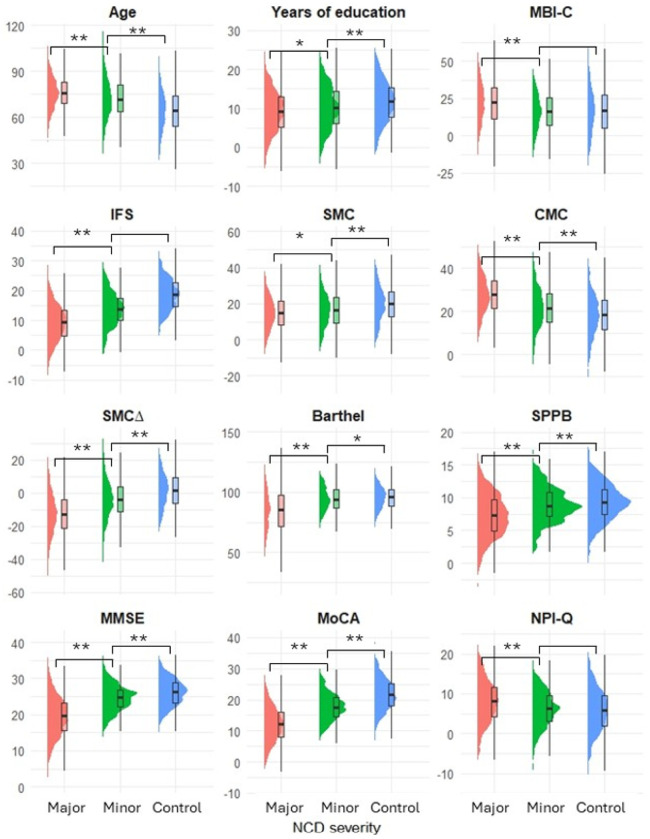
Differences between variables across neurocognitive disorder severity SMC: Subjective memory complaint; CMC: Caregiver Memory Complaint;NCD: neurocognitive disorder; MMSE: Mini-Mental State examination; MoCA: Montreal Cognitive Assessment; NPI-Q: Neuropsychiatric Inventory Questionnaire; MBI-C: Mild Behavioral Impairment Checklist; IFS: Ineco Frontal Screening.

Patients had a mean age of 71.71 years (SD: 13.53), and 58.87% were women (n = 1,974). Regarding memory complaints, the scores from SMC were lower than the CMC in major and mild cognitive disorders (15.01 vs. 27.98 and 16.5 vs. 20.97, respectively), but higher in the control group (20.03 vs. 18.37). Additional patient characteristics are detailed in Table 1. When comparing NCD severity, statistically significant differences were observed, with higher values in major NCD for all variables except sex, as well as the prevalence of metabolic and neurological comorbidities. All differences and p-values are presented in Fig. 2 and Annex 2.

Before the linear regression model development, we did not find a significant interaction between the independent variables in any model, so we included all variables in the forward models. The adjusted R2 for each model are: 8.9% for SMC, 35.4% for CMC, and 30.5% for SMCΔ, all overall models were statistically significant. The most parsimonious models for each outcome in all populations are the following: 1) considering SMC as the outcome (R2 = 9.6%, p < 0.001), the final predictive variables are age (β= −0.159, p < 0.001), higher NCD severity (β = 1.804, p < 0.001), years of education (β= −0.192, p < 0.001), neurologic comorbidity (β = 0.912, p < 0.001), SPPB (β= −0.172, p < 0.001), MoCA (β= −0.178, p < 0.001), NPI-Q (β = 0.146, p < 0.001) and IFS (β < 0.001, p = 0.102). 2) Considering CMC as the outcome (R2 = 36%, p < 0.001), the definitive predictive variables are higher NCD severity (β= −1.937, p < 0.001), years of education (β = 0.155, p = 0.001), psychiatric comorbidity (β= −0.05, p = 0.029), MMSE (β= −0.202, p = 0.019), MoCA (β= −0.312, p < 0.001) and MBI-C (β = 0.209, p < 0.001). 3) Considering SMCΔ as the outcome (R2 = 30.5%, p < 0.001), the definitive predictive variables are age (β= −0.172, p < 0.001), higher NCD severity (β = 3.74, p < 0.001), years of education (β= −0.347, p < 0.001), psychiatric comorbidity (β = 0.963, p = 0.206), Barthel (β= −0.039, p = 0.205), MMSE (β = 0.328, p = 0.003) and MBI-C (β= −0.212, p < 0.001). These models are displayed in Table 2. When stratifying by neurocognitive impairment severity, all models were statistically significant and reflected p values < 0.05 in most of the independent variables and conserved the association direction. See annex 3, 4 and 5.

**Figure 3:**
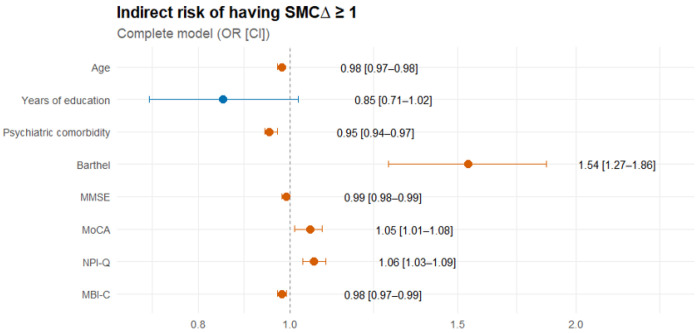
Forest plot of measures of association regarding the indirect risk of having SMCΔ ≥ 1 OR: Odds ratio; CI: Confidence interval; SMC: Subjective memory complaint; MMSE: Mini-Mental State examination; MoCA: Montreal Cognitive Assessment; NPI-Q: Neuropsychiatric Inventory Questionnaire; MBI-C: Mild Behavioral Impairment Checklist.

Concerning the indirect risk of having SMCΔ ≥1, the logit model showed a statistically significant association. These were proportional to Barthel, MoCA, and NPI-Q, increasing the chance of presenting SMCΔ ≥ 1 in 53.7%, 4.8%, and 6.1%, respectively. The other variables showed an inverse relationship, i.e. higher CMC than SMC. Finally, the most parsimonious model (AIC: 1891.1) included only years of education (OR = 1.029), MoCA (OR = 1.073), and NPI-Q (OR = 1.035) (p < 0.05) (Fig. 3, Annex 6).

## Discussion

In this study, we explored the association between SMC and CMC with sociodemographic, cognitive, neuropsychiatric, and clinical variables in a cohort from a Colombian memory clinic. We also introduced a novel metric, SMCΔ—the discrepancy between SMC and CMC—as a proxy for potential anosognosia (low SMC) or caregiver overestimation (high CMC). Our findings indicate that the variability in both patient- and caregiver-reported memory complaints is influenced by cognitive performance, functional status, neuropsychiatric symptoms, and sociodemographic factors. Specifically, (A) CMC and SMCΔ demonstrated stronger associations with cognitive, functional, neuropsychiatric, and sociodemographic variables. (B) CMC was inversely associated with MMSE scores, whereas both SMC and SMCΔ showed a positive association. MoCA scores were inversely associated with both SMC and CMC. (C) Neuropsychiatric symptoms, as measured by the NPI-Q, were positively associated with memory complaints, a pattern also observed for the MBI-C, except in the case of SMC. (D) Functional measures showed statistically significant associations only with SMC, reflecting an inverse relationship. (E) Neurological conditions were primarily associated with SMC, while psychiatric conditions showed stronger associations with both CMC and SMC. (F) Higher scores on the Barthel Index, MoCA, and NPI-Q increased the odds of presenting with higher SMC compared to CMC.

Our findings provide valuable evidence from basic clinical assessments, demonstrating a low-cost, scalable approach to support early detection of cognitive impairment—particularly relevant in health systems with limited access to advanced diagnostics. This aligns with global public health priorities advocating for improved dementia diagnosis and care in low- and middle-income countries (LMICs).^3,4,37,38^ Our results also reinforce the importance of incorporating memory complaints into routine evaluations as an initial screening step, which can be combined with periodic cognitive assessments to enhance diagnosis and follow-up, especially in high-prevalence regions.^10^ Promoting sustainable strategies like annual cognitive check-ups could further improve longitudinal monitoring and care planning.^39^ Our study supports the evaluation of individuals without overt impairment, contributing to more precise and personalized care across the neurocognitive continuum. The integration of subjective memory complaints and their discrepancies (SMCΔ) in clinical assessment of cognitive functioining offers a cost-effective, reproducible method to track cognitive changes during routine consultations. Self- and proxy-reports will help clinicians to better detect early decline, stratify risk, and guide timely interventions—even in resource-constrained environments.

This study has important clinical implications, as it supports the evaluation of individuals without overt cognitive impairment, thus facilitating more precise and personalized care across the continuum of neurocognitive disorders. With the use of patient- and caregiver-reported memory complaints, clinicians could detect in a more opportune and accurate manner those subtle changes that are present in early disease stages, and guide timely interventions even in resource-limited settings.

Regarding sociodemographic features, our results align with previous evidence showing that lower education levels are correlated with individuals reporting higher SMC and CMC.^40^ These findings underscore the differential impact of cognitive reserve on the expression of SMC and its association with dementia risk. Notably, studies have shown that among individuals with higher education, SMC may predict AD risk at nearly twice the rate observed in those with lower education levels.^41^ These findings suggest that educational background is a critical factor in evaluating the clinical relevance of memory complaints. This consideration is particularly crucial in regions such as Latin America, where lower levels of formal education are more prevalent, thereby amplifying the need to contextualize cognitive assessments within the broader sociocultural environment.^42^ However, our study only considered the patient’s education level and future research should examine the caregiver’s educational background to determine its influence on SMC and CMC, therefore, these results should be applied carefully, and future studies should consider this possible interaction.

The relationship between memory complaint and MMSE and MoCA in our results contrasts with previous literature, where SMC has been linked to poorer neuropsychological performance, particularly in visual and verbal memory tasks.^43,44^ Previous studies have questioned whether SMC reflects actual cognitive decline, as findings have been inconsistent.^14^ SMC has shown to have a differential effect according to the type of memory, with impaired performance in immediate and short-term memory.^45^ In contrast, SMC is directly related to global cognition and other cognitive domains such as executive function, attention, and processing speed.^7,14,46^ Although the MMSE and MoCA are widely used, they may not fully capture subtle impairments outside the Alzheimer’s disease spectrum. However, their utility may improve when interpreted in conjunction with memory complaints, especially for differentiating between diagnostic categories.

Our findings coincide with a previous report^33^ as revealed associations between higher neuropsychiatric symptoms and major CMC. It suggests that CMC may serve as a more objective marker of decline, as informant-reported cognitive difficulties tend to have higher prognostic value compared to patient’s complaints^47^, that can be explained due to anosognosia.^48^ However, this could also be because of the caregiver burden, which has been related to the presence and severity of behavioral symptoms,^49,50^ leading to an overestimation of reporting such alterations. It enhances the importance of SMCΔ and its possible capability to reduce bias.

Prior studies have shown that affective states predominantly influence SMC,^9^ showing higher scores of SMC in people with anxious or depressive symptoms.^51^ These findings are aligned with ours, since psychiatric conditions were strongly linked to CMC and SMCΔ, while neurological conditions were associated only with SMC. This could be due to biased beliefs, such as poor perceived health or cognitive performance, and multiple somatic and cognitive complaints.^9^ These effects may extend to both patients and caregivers, influencing their subjective reports, thus explaining the effect shown in our study. While our study did not differentiate between specific psychiatric disorders because we wanted to look for a global panorama, our findings emphasize the need for psychiatric evaluation in memory clinics, as neuropsychiatric symptoms can modulate both patient and caregiver perceptions of cognitive decline, along with other brain-health variables such as social activities or interpersonal interactions. Future studies should delve deeper into different etiologies and assess specific patterns.

Exploration of SMCΔ, as a measure of discrepancies between patient-caregiver, along with its associated factors, as our results indicate that higher Barthel, MoCA, and NPI-Q scores are key predictors of SMCΔ ≥ 1, a higher SMC or patient overestimation. The associated variables reflect essential components of memory clinic assessments: cognitive reserve (education), cognitive performance (MoCA), and neuropsychiatric symptoms (NPI-Q). While our findings suggest that SMCΔ could serve as a valuable clinical tool, its interpretation should be integrated with these other measures, emphasizing the need for interdisciplinary collaboration, considering that this is the first report of this approach. Future studies should use this metrics with longitudinal approaches to identify the source of the highest memory complaint and the magnitude of its discrepancy, as well as identify the prediction capacity of SMCΔ.

The strengths of this study come from the measurement of SMC, since the validated scale used considers SMC and CMC, and the interdisciplinary and systematic evaluation in our center, which allows us to have a clearer picture of the interaction between different factors that can be reflected in variables that are easy to measure, such as SMC. It is also important to highlight the large sample size, and that it comes from the largest real-world data sample of a memory clinic in LA. Additionally, SMC has been widely used in research, yet it has been measured in different ways,^52^ therefore, the use of a systematized method, as well as a contrast between patient and caregiver, gives a more objective meaning to this measure.

This article has some limitations. First, the study’s cross-sectional nature does not allow us to infer temporality and causality in reported associations. However, even if it was not the objective of our study, future research should evaluate temporality and include a follow-up. Also, social support could mediate the impact of caregiver burden and other social determinants of health, so it is necessary to carry out new studies evaluating the role of more sociodemographic variables and caregiver burden. Moreover, there are certain difficulties in the measurement of CMC since the degree of closeness and knowledge of the family member regarding the patient’s situation was not specified in the statistical analyses. Further, we did not carry out subanalyses considering cognitive or neuropsychiatric domains due to our focus on an overall pattern of cognitive, behavioral and functional performance.

Importantly, we addressed a critical limitation in the use of memory complaints—namely, the risk of subjective under- or overestimation. By combining both patient- and caregiver-reported complaints and introducing the SMCΔ metric, we were able to reduce potential biases and better capture discrepancies that may reflect anosognosia or caregiver overreporting. This integrative approach provides a more nuanced understanding of cognitive concerns in real-world settings and supports the development of more equitable and effective screening models.

## Conclusion

Our findings highlight the association between patient- and caregiver-reported SMC with cognitive, functional, and neuropsychiatric factors, and for assessing the clinical relevance of different scores according to functional, cognitive, and behavioral factors. Additionally, we introduce SMCΔ as a novel approach to capturing discrepancies between self- and caregiver-reported complaints, potentially improving the early detection of cognitive decline and contributing to a deeper understanding of cognitive complaints, along with their underlying mechanisms and associated factors. By incorporating both patient- and caregiver-reported complaints, SMCΔ provides a more comprehensive evaluation, potentially guiding earlier interventions and personalized treatment strategies to slow disease progression and reduce caregiver burden.

This study suggests that SMCΔ may serve as a meaningful marker for diagnosing and monitoring cognitive deterioration across different disease stages, as well as to differentiate it from differential diagnosis. Moreover, it offers a simple, accessible tool that could facilitate clinical assessments, particularly in low- and middle-income countries (LMICs), where advanced diagnostic resources may be limited.

Authors participation: FBR and JMS generated the research idea, FBR, JCC, JMS and HS-G designed the project. FBR and SSL drafted the article. FBR and JCC implemented the models. HS-G supervised the project. The rest of the authors provided critical revisions. All authors reviewed and accepted the manuscript.

## Supplementary Material

This is a list of supplementary files associated with this preprint. Click to download.

• SupplementaryMaterial.docx

## Figures and Tables

**Table 1:** Patients’ characteristics, mean (SD)

n	3354
Age	71.71 (13.53)
Female n (%)	1974 (58.87)
Years of education	10.20 (5.74)
SMC	16.86 (10.47)
CMC	23.75 (10.60)
SMCΔ	−6.89 (13.74)
Metabolic disease (%)	63.25
Neurologic comorbidity (%)	27.23
Psychiatric comorbidity (%)	22.89
Barthel	88.26 (16.86)
SPPB	7.93 (3.36)
MMSE	21.38 (5.69)
MoCA	14.66 (6.74)
NPI-Q	7.39 (5.53)
MBI-C	20.49 (15.48)
IFS	11.71 (6.89)

SD: standard deviation; SMC: Subjective memory complaint; CMC: Caregiver Memory Complaint; MMSE: Mini-Mental State examination; MoCA: Montreal Cognitive Assessment; NPI-Q: Neuropsychiatric Inventory Questionnaire; MBI-C: Mild Behavioral Impairment Checklist; IFS: Ineco Frontal Screening

**Table 2.** Association’s model for Subjective Memory Complaint in all population

Measure						
	CMC		SMC		SMCΔ	
	R2	AIC	R2	AIC	R2	AIC
	0.354	5471	0.089	5685	0.305	6151

	**β (SE)**	**p value**	**β (SE)**	**p value**	**β (SE)**	**p value**

Age	0.013	0.56	−0.159	<0.001	−0,172	<0.001
Male	−0.137	0.792	0.567	<0.001	0.704	0.304
NCD severity	−1.937	<0.001	1.804	<0.001	3.74	<0.001
Years of education	0.155	0.001	−0.192	<0.001	−0.347	<0.001
Metabolic disease (%)	−0.112	0.828	0.135	<0.001	0.247	0.715
Neurologic comorbidity (%)	−0.05	0.93	0.912	<0.001	0.963	0.206
Psychiatric comorbidity (%)	0.403	0.029	−0.177	<0.001	−0.579	0.016
Barthel	0.018	0.452	−0.021	<0.001	−0.039	0.205
SPPB	−0.089	0.376	−0.172	<0.001	−0.084	0.524
MMSE	−0.202	0.019	0.127	<0.001	0.328	0.003
MoCA	−0.312	<0.001	−0.178	<0.001	0.133	0.191
NPI-Q	0.081	0.308	0.146	<0.001	0.065	0.53
MBI-C	0.209	<0.001	−0.002	<0.001	−0.212	<0.001
IFS	− <0.001	0.968	− <0.001	0.102	− <0.001	0.183
	**R2**	**AIC**	**R2**	**AIC**	**R2**	**AIC**
	0.357	5457	0.096	5677	0.305	6144

**Final models**	**β (SE)**	**p value**	**β (SE)**	**p value**	**β (SE)**	**p value**

Age			−0.159	<0.001	−0.174	<0.001
NCD severity	−1.992	<0.001	1.856	<0.001	3.854	<0.001
Years of education	0.148	<0.001	−0.182	<0.001	−0.324	<0.001
Neurologic comorbidity (%)			0.989	<0.001		
Psychiatric comorbidity (%)	0.408	<0.001			−0.579	0.016
Barthel					−0.052	0.041
SPPB			−0.215	<0.001		
MMSE	−0.198	<0.001			0.444	<0.001
MoCA	−0.323	<0.001	−0.095	<0.001		
NPI-Q			0.149	<0.001		
MBI-C	0.23	<0.001			−0.191	<0.001
IFS			− <0.001	0.085		

* All models are p < 0,001; R2: Adjusted R-squared; AIC: Akaike Information Criterion; β: Beta coefficient; SE: standard error; SMC: Subjective memory complaint; CMC: Caregiver Memory Complaint; NCD: neurocognitive disorder; MMSE: Mini-Mental State examination; MoCA: Montreal Cognitive Assessment; NPI-Q: Neuropsychiatric Inventory Questionnaire; MBI-C: Mild Behavioral Impairment Checklist; IFS: Ineco Frontal Screening

## Data Availability

The datasets generated and/or analysed during the current study are not publicly available due to they are sensitive data from our clinic, but are available from the corresponding author on reasonable request.
